# Prevalence, risk factors and disability associated with fall-related injury in older adults in low- and middle-incomecountries: results from the WHO Study on global AGEing and adult health (SAGE)

**DOI:** 10.1186/s12916-015-0390-8

**Published:** 2015-06-23

**Authors:** Jennifer Stewart Williams, Paul Kowal, Heather Hestekin, Tristan O’Driscoll, Karl Peltzer, Alfred Yawson, Richard Biritwum, Tamara Maximova, Aarón Salinas Rodríguez, Betty Manrique Espinoza, Fan Wu, Perianayagam Arokiasamy, Somnath Chatterji, SAGE collaborators

**Affiliations:** Research Centre for Gender, Health and Ageing, Faculty of Health, University of Newcastle, Newcastle, Australia; Epidemiology and Global Health, Umeå University, Umeå, Sweden; Surveys, Measurement and Analysis Unit, World Health Organization, Geneva, Switzerland; University of Southern California, Los Angeles, USA; Midwestern University Chicago College of Pharmacy, Chicago, USA; Human Sciences Research Council, Pretoria, South Africa; University of Limpopo, Turfloop, Sovenga, South Africa; Mahidol University, Salaya, Phutthamonthon, Nakhonpathom Thailand; Department of Community Health, University of Ghana, Accra, Ghana; National Research Institute of Public Health (FSBI, RAMS), Moscow, Russian Federation; Center for Evaluation Research and Surveys, National Institute of Public Health, Cuernavaca, Morelos Mexico; Shanghai Municipal Center for Disease Control and Prevention, Shanghai, China; International Institute of Population Sciences, Mumbai, India

**Keywords:** Falls, Injuries, Disabilities, Chronic conditions, Developing countries, Ageing

## Abstract

**Background:**

In 2010 falls were responsible for approximately 80 % of disability stemming from unintentional injuries excluding traffic accidents in adults 50 years and over. Falls are becoming a major public health problem in low- and middle-income countries (LMICs) where populations are ageing rapidly.

**Methods:**

Nationally representative standardized data collected from adults aged 50 years and over participating in the World Health Organization (WHO) Study on global AGEing and adult health (SAGE) Wave 1 in China, Ghana, India, Mexico, the Russian Federation and South Africa are analysed. The aims are to identify the prevalence of, and risk factors for, past-year fall-related injury and to assess associations between fall-related injury and disability. Regression methods are used to identify risk factors and association between fall-related injury and disability. Disability was measured using the WHO Disability Assessment Schedule Version 2.0 (WHODAS 2.0).

**Results:**

The prevalence of past-year fall-related injuries ranged from 6.6 % in India to 1.0 % in South Africa and was 4.0 % across the pooled countries. The proportion of all past-year injuries that were fall-related ranged from 73.3 % in the Russian Federation to 44.4 % in Ghana. Across the six countries this was 65.7 %. In the multivariable logistic regression, the odds of past-year fall-related injury were significantly higher for: women (OR: 1.27; 95 % CI: 0.99,1.62); respondents who lived in rural areas (OR: 1.36; 95 % CI: 1.06,1.75); those with depression (OR: 1.43; 95 % CI: 1.01,2.02); respondents who reported severe or extreme problems sleeping (OR: 1.54; 95 % CI: 1.15,2.08); and those who reported two or more (compared with no) chronic conditions (OR: 2.15; 95 % CI: 1.45,3.19). Poor cognition was also a significant risk factor for fall-related injury.

The association between fall-related injury and the WHODAS measure of disability was highly significant (*P*<0.0001) with some attenuation after adjusting for confounders. Reporting two or more chronic conditions (compared with none) was significantly associated with disability (*P*<0.0001).

**Conclusions:**

The findings provide a platform for improving understanding of risk factors for falls in older adults in this group of LMICs. Clinicians and public health professionals in these countries must be made aware of the extent of this problem and the need to implement policies to reduce the risk of falls in older adults.

**Electronic supplementary material:**

The online version of this article (doi:10.1186/s12916-015-0390-8) contains supplementary material, which is available to authorized users.

## Background

Falls are a leading cause of unintentional injury and premature death worldwide [1]. In 2010, falls were responsible for approximately 80 % of disability stemming from unintentional injuries excluding traffic accidents in adults aged 50 years and over [2]. Globally falls are a major public health concern for older adults, and with the growing numbers of older people in populations in all parts of the world, research is urgently needed in order to establish effective policies to reduce risk.

The physical and mental changes associated with advancing age and frailty increase the risk of fall-related injury. Every year one-third of community-dwelling older adults fall. About 10–15 % subsequently endure an injury [3–5]. Approximately 3 % of community-dwelling older adults sustain significant injuries due to falls in any given year [6], resulting in substantial costs to individuals and society [1, 7–10].

Already over 70 % of the world’s older population live in developing countries. The proportion will increase in coming decades due to increasing longevity in all regions of the world [11]. One of the consequences of this demographic change is that a greater share of the burden of morbidity and mortality due to falls and other chronic conditions will occur in low- and middle-income countries (LMICs). Differences are already evident. In 2010, for example, years lived with disability (YLDs) due to reported falls were 631.2 per 100,000 (population) in India and 674.4 per 100,000 in China, compared with 472.2 per 100,000 in the United States [2]. In that year, the global share of YLDs due to falls in adults aged 50 to 59 years was 66 % in developing countries and 34 % in high-income developed countries [1].

There are several hundred possible risk factors for falls in older adults [3, 12]. They include: older age, female gender, physical frailty, muscle weakness, unsteady gait and balance, impaired cognition [4, 10, 12–17] and depressive symptoms [18–20]. The risk of falling increases with age and with a higher disease burden from chronic conditions such as cardiovascular disease, arthritis and diabetes [21–23]. Nutritional deficiency, poor sleep patterns and visual impairment are also associated with increased risk of falling [20, 24, 25].

Social and economic factors, low income, low education and inadequate housing are risk factors for falls [9, 26]. Environmental features are not risk factors per se, but they encapsulate the interaction between the individual and his or her environment in ways that can precipitate falls. Home hazards, such as slippery floors and poor lighting, and features of the public environment, such as poor building design and uneven sidewalks, increase the risk of falls in older adults [9]. Some low-cost interventions have been identified for falls prevention, yet implementation is occurring mostly in high-income countries [17, 27].

Evidence of falls in older adults in LMICs is sparse, and there is a lack of standardized terminology and definition. Methodological and sampling differences make it difficult to generalize across diverse settings and populations [23, 28]. A review of studies of falls in India showed annual fall rates for older adults of between 14 and 51 % [22]. Studies in China, Hong Kong, Macao, Singapore and Taiwan [12] reported annual rates of falls for adults aged 60 years and over of between 14.7 and 34 %. Falls prevention is not given a high policy priority in many developing countries, in part because of the lack of valid generalizable evidence [9]. Governments in LMICs urgently require data and evidence to develop and integrate falls prevention into their policy and planning frameworks [1, 2, 9, 27–30].

This study addresses a gap in epidemiological falls research in developing countries. Cross-sectional data from WHO’s Study on global AGEing and adult health (SAGE) Wave 1 are analysed to investigate determinants and conditions associated with fall-related injury and disability in adults aged 50 years and over [9, 27, 29]. The data are drawn from nationally representative cohorts of adults in China, Ghana, India, Mexico, the Russian Federation and South Africa. The aims of this study are to: identify the prevalence of self-reported past-year fall-related injury; describe risk factors associated with past-year fall-related injury; and assess associations between past-year fall-related injury and disability.

## Methods

### Ethics statement

The SAGE study was approved by the following bodies: the Ethics Review Committee, World Health Organization, Geneva, Switzerland; Ethics Committee, Shanghai Municipal Centre for Disease Control and Prevention, Shanghai, China; Ethical Committee, University of Ghana Medical School, Accra, Ghana; Institutional Review Board, International Institute of Population Sciences, Mumbai, India; Ethics Committee, National Institute of Public Health, Cuernavaca, Mexico; Ethics Committee, School of Preventive and Social Medicine, Russian Academy of Medical Sciences, Moscow, Russia; and the Research Ethics Committee, Human Sciences Research Council, Pretoria, South Africa. This approval covered all procedures undertaken as part of the study. Written informed consent was freely obtained from each individual participant. Confidential records of participants’ consent are maintained by SAGE country teams.

### Study design

SAGE is a longitudinal study with nationally representative samples of adults in China, Ghana, India, Mexico, the Russian Federation and South Africa. Wave 1 (2007–2010) data were collected via in-person structured interviews. One household questionnaire was completed per household, and all individuals aged 50 years and over in selected households were invited to participate [31].

SAGE used a multistage stratified random sampling in all six countries to ensure that aggregate results within a country are nationally representative. Stratification was based on the size of the first unit of selection (for example, region/province) and type of locality (for example, urban/rural). A probability proportional to size (PPS) method was used to select the primary sampling units, and households were selected randomly within these units [32]. All adults aged 50 and over in a household were selected. Post-stratification weights were then generated to adjust for the age and sex population distributions of the respective countries at the time of survey. Pooled weights, based on WHO’s World Standard Population [33] and estimates from the United Nations Statistical Division [34, 35], were applied to adjust for age and sex differences between countries. Additional details about SAGE are provided elsewhere [31].

### Past-year fall-related injury

A fall is defined here as an event which results in a person coming to a rest inadvertently on the ground or floor or other lower level [9]. The variable ‘fall-related injury’ was derived from responses to questions from the WHO guidelines on injuries which were included in the SAGE individual questionnaire [36]. The question was asked: “In the last 12 months, have you had any other event (other than a road traffic accident) where you suffered from bodily injury?” The follow-up question for those who answered ‘yes’ was: “What was the cause of this injury?” A list of possible responses was offered during the interviews (struck by a person or object, stabbed, gun shot, animal bite, electric shock or fall). This refers to ‘unintentional’ injuries only. When respondents explicitly reported a fall as the cause of their injury, it was defined as a ‘fall-related injury’. The variable ‘past-year fall-related injury’ (no versus yes) refers to the occurrence (by self-report) of any fall-related injury in the year prior to the survey interview.

### Disability

Disability was measured using the cross-culturally validated 12-item version of the WHO Disability Assessment Schedule Version 2.0 (WHODAS 2.0) encompassing six domains measuring functioning and disability [37]. The 12 items included in the scale refer to difficulty in functioning in the past 30 days such as with bathing and dressing oneself, learning a new task, participation in community activities and household chores. Responses were measured on a Likert scale, ranging from ‘no difficulty’ to ‘severe difficulty’ or ‘cannot carry out the activity’. Responses to the questions were summed to obtain a composite score which was transformed to a 0–100 scale, with a higher score indicating a higher level of disability [38, 39].

### Covariates

Using the literature on falls as a guide [3, 4, 9, 10, 12–20, 22–25, 40], commonly cited risk factors were selected as potential covariates and then identified in the SAGE dataset. Categorical risk factor variables in the SAGE individual questionnaire were: sex (male versus female); age in years (50–59; 60–69; 70–79, and 80+); nutrition/diet (sufficient intake of fruit and vegetables >=5 servings daily versus insufficient intake <5 servings daily [41, 42]; sleep (no serious or extreme problems versus severe or extreme sleep problems in the previous 30 days) [43, 44]; symptom-based depression diagnosis (no versus yes); self-reported cataracts (no versus yes); and residence (urban versus rural). Data on weight in kilograms and height in metres was used to derive body mass index (BMI) [45]. The WHO guidelines were used to create BMI categories: underweight less than 18.50 kg/m^2^; normal weight 18.50–24.99 kg/m^2^; pre-obese 25.00–29.99 kg/m^2^; obese greater than or equal to 30.00 kg/m^2^ [46, 47]. Given evidence that older adults with chronic conditions are at greater risk of undergoing falls [3, 9, 17, 20, 23], respondents were classified according to a chronic disease count of up to five possible chronic conditions: depression, arthritis, angina, asthma and diabetes. Depression, angina and arthritis were derived from validated symptom-based algorithms [48], and diabetes was based on self-reported responses. A hierarchical ordered probit model was used to develop an index of household asset ownership of durable goods (such as chairs, tables, cars, television, telephone and washing machine), dwelling characteristics (such as type of floors, walls, and cooking stove), access to services such as improved water and sanitation, electricity and type of cooking fuel used in the household [49, 50]. Country-specific ‘wealth quintiles’ were generated from this index. The wealth quintiles provide an alternate measure of income with set incremental levels of assets that are less likely to be biased by respondent inconsistencies in income reporting [49].

Continuously measured risk factor variables in the individual questionnaire were: cognition, grip strength and gait speed [10, 12, 13, 15, 51]. A cognition score (higher meaning better) was computed by summing scores on tests of verbal recall, digit span (forward and backwards) and verbal fluency. Mean grip strength (in kilograms) was generated by averaging the results of two attempts with a dynamometer in each hand. Gait (walking) speed (in metres/second) at normal/usual pace was measured over a four-metre length. These scores were standardized to ensure better comparability.

Many falls in community-dwelling older adults occur in the home, and there are many ‘home hazards’ that precipitate falls [4, 9, 17, 20]. Potential risk factors identified in the household questionnaire dataset were: dwelling characteristics (hard material floor versus earth floor), and water source (water inside the home versus water outside the home).

### Descriptive characteristics

In addition to sex, age, residence and wealth quintile, the study population is also described by marital status (never married versus married or cohabiting versus separated, divorced, or widowed); work status (currently working for pay versus not currently working for pay versus never worked for pay); and education level (no formal schooling versus completed primary school versus completed secondary/high school versus completed university/college). A classification scheme based on the 1997 International Standard Classification of Education [52] was used in each country to map the highest completed level of education [26].

### Statistical analyses

The study population is comprised of SAGE respondents aged 50 years and over with completed interviews. Respondents with complete data on sex, age, place of residence, marital status, work status, wealth and education are described by these characteristics.

Prevalence is measured as the cross-sectional percent likelihood of an individual self-reporting the occurrence of one or more (‘any’) injuries or fall-related injuries in the year prior to the SAGE survey interview. Three sets of past-year prevalence measures are given: the proportion of respondents with any reported past-year injuries; the proportion of respondents with any reported past-year fall-related injuries; and the proportion of respondents with any reported past-year injuries who reported any past-year fall-related injuries.

Individual and household datasets were merged (many to one) using common identifiers. The literature was used to inform candidate variables for testing in the logistic regression of past-year fall-related injury and the linear regression of the WHODAS disability score. Variables chosen were those that were commonly cited and also identifiable in the SAGE individual and household questionnaires.

The SAGE individual and household questionnaire records were linked for regressions in the pooled dataset covering the six SAGE countries. We undertook multivariable logistic regression to describe risk factors associated with past-year fall-related injury (outcome) and multivariable linear regression of past-year fall-related injury (exposure) and disability (outcome). A six-category country variable, with China as the reference group, was included in each of the regressions to allow for between-country differences. Bivariate analyses were used first to screen candidate variables (*P*<0.05) before proceeding to model building. Variables were checked for correlation and collinearity.

In the logistic regression, models were tested by inspecting the estimated coefficients using the Wald statistic. The likelihood ratio test (*P*<0.05) was used to compare and evaluate each model with the next. In the linear regression, model comparisons were made using the adjusted R*-*squared statistic, while also taking into account the statistical significance of the individual covariates at each step. Pairs of covariates were assessed for possible interactions. Where interactions were statistically significant (*P*<0.05), models were stratified to investigate effect modification. Multicollinearity was tested using the variance inflation factor (VIF) statistic, which is reported in the tables with model fit statistics. The VIF estimates how much of the variance is due to collinearity with other predictors. Model assumptions were checked.

All analyses included survey weights to produce nationally representative country and multi-country samples. Age and sex standardizations were carried out in the pooled dataset covering the six countries. Statistical significance was set at *P*<0.05. STATA Version 11 (StataCorp, College Station, TX, 2009) was used for all statistical analyses.

## Results

### Socio-demographic characteristics

Table 1 describes socio-demographic characteristics of the study population. The denominator is all SAGE respondents aged 50 years and over with complete data on sex, age, place of residence, marital status, work status, wealth and education. In the pooled sample (N=32,663), there was a higher proportion of women than men (51 % versus 49 %) as there was also in the individual countries, except for Ghana (48 %) and India (49 %). In the Russian Federation, the sample consisted of 61 % women and 39 % men. In the pooled countries, almost 5 % of respondents were aged 80 years and over; however, Ghana purposely oversampled the oldest old adult population resulting in almost 10 % of the sample aged 80 years and over. The distribution of the population between urban and rural locations in the pooled dataset was 44 % urban versus 56 % rural. Approximately 45 % of respondents reported that they were currently working for pay. In Russia, just 2 % reported not having formal education, with higher percentages in the other five countries. Russia had the highest proportion of respondents with completed university or college studies (18 %).

**Table 1:** Percentage distribution of socio-demographic characteristics of adults aged 50 years and over, by country and pooled, SAGE Wave 1, 2007–2010

	China	Ghana	India	Mexico	Russia	S. Africa	Pooled
Observations (N)	12,852	4,236	6,521	2,200	3,736	3,123	32,663
Sex	%	%	%	%	%	%	%
Men	49.8	52.3	51.0	46.7	39.0	40.2	48.8
Women	50.2	47.7	49.0	53.4	61.0	59.8	51.2
Age group (years)							
50–59	45.2	39.8	48.6	49.2	45.1	50.0	49.8
60–69	31.9	27.5	30.9	25.8	24.6	30.6	28.7
70–79	18.6	23.0	16.0	17.8	21.8	14.1	16.1
80+	4.3	9.7	4.5	7.3	8.5	5.4	5.4
Residence							
Urban	47.7	40.9	29.0	78.4	72.8	64.7	44.2
Rural	52.3	59.1	71.0	21.6	27.2	35.3	55.8
Marital status							
Never married	1.0	1.2	0.7	7.0	2.7	14.9	1.3
Married/cohabiting	85.4	59.2	77.0	73.0	58.3	52.5	80.3
Separated/divorced/widowed	13.6	39.5	22.3	20.0	39.0	32.6	18.4
Work status							
Currently working for pay	43.6	69.0	43.2	37.4	40.0	30.4	44.5
Not currently working for pay	47.5	29.4	29.9	24.0	59.2	55.8	41.3
Never worked	8.9	1.6	26.9	38.5	0.8	13.9	14.2
Wealth quintile							
Lowest	16.1	18.2	18.2	15.2	16.2	20.8	16.8
Second	18.1	19.0	19.5	25.0	19.6	20.3	18.8
Third	20.5	20.7	18.8	16.5	19.1	19.1	19.6
Fourth	23.4	20.8	19.6	16.7	20.6	19.3	21.9
Highest	21.9	21.4	23.9	26.6	24.4	20.5	23.0
Education level							
No formal schooling	41.4	64.2	61.2	55.6	2.0	48.5	44.3
Completed primary school	21.3	11.0	14.8	24.0	5.5	22.7	18.2
Completed secondary school	32.7	21.2	18.8	12.3	74.2	23.0	31.8
Completed university/college	4.6	3.6	5.1	8.1	18.3	5.8	5.7

S. Africa=South Africa; Russia=Russian Federation. Denominator comprises complete cases. Percentages include survey weights (countries and pooled). N=observations (not weighted)

Table 2 compares different self-reported measures of unintentional injury prevalence. The prevalence of respondents with past-year injuries (excluding traffic accidents) was 6.0 % in the pooled countries, with the highest proportion (9.1 %) in India and the lowest in South Africa (1.3 %). The prevalence of past-year fall-related injuries ranged from 6.6 % in India to 1.0 % in South Africa and was 4.0 % in the pooled countries. The proportion of past-year injuries that were fall-related was 65.7 % over all countries, ranging from 73.3 % in Russia to 44.4 % in Ghana.

**Table 2:** Past-year prevalence of all self-reported unintentional injuries and fall-related injuries, adults aged 50 years and over, by country and pooled, SAGE Wave 1, 2007–2010

Country	% respondents (N) reporting past-year injuries	% respondents (N) reporting past-year fall-related injuries	% respondents with reported past-year injuries (N) that were reported as fall-related
China	5.1 % (N=13,158)	3.1 % (N=13,158)	60.6 % (N=595)
Ghana	5.7 % (N=4,305)	2.6 % (N=4,305)	44.4 % (N=256)
India	9.1 % (N=6,560)	6.6 % (N=6,560)	72.3 % (N=587)
Mexico	4.2 % (N=2,318)	2.8 % (N=2,318)	67.2 % (N=118)
Russian Federation	3.3 % (N=3,763)	2.4 % (N=3,763)	73.3 % (N=142)
South Africa	1.3 % (N=3,836)	1.0 % (N=3,836)	67.0 % (N=51)
Pooled	6.0 % (N=33,922)	4.0 % (N=33,922)	65.7 % (N=1,750)

Past-year refers to self-report of injuries in the year prior to the survey interview. Percentages include survey weights (countries and pooled). N=observations (not weighted). Road traffic accidents excluded

Table 3 shows the weighted results of the crude and multivariable logistic regression of factors associated with past-year fall-related injury. The unweighted results of the multivariable logistic regression are given in Additional file 1: Appendix 1. Variables tested as possible risk factors but not included here because of non-significance in bivariate association with past-year fall-related injury were: BMI, nutrition, wealth quintile and gait speed. In the multivariable model, the odds of past-year fall-related injury were almost 30 % higher for women than men (OR: 1.27; 95 % CI: 0.99,1.62). Respondents who lived in rural areas had 36 % higher odds of reporting past-year fall-related injury (OR: 1.36; 95 % CI: 1.06,1.75) compared with those who lived in urban areas, and respondents with symptom-based depression had 43 % higher odds of reporting past-year fall-related injury (OR: 1.43; 95 % CI: 1.01,2.02). Respondents who reported severe or extreme problems sleeping had over 50 % higher odds of reporting past-year fall-related injury (OR: 1.54; 95 % CI: 1.15,2.08), and poor cognition (as a continuous variable) was also a risk factor for past-year fall-related injury. In the weighted multivariable logistic regression, a one-unit average increase in the cognition score was associated with a 15 % lower likelihood of past-year fall-related injury. The presence of multiple chronic conditions was significant in association with past-year fall-related injuries. Respondents with two or more chronic conditions were more than twice as likely to report past-year fall-related injuries compared with respondents with no reported chronic conditions (OR: 2.15; 95 % CI: 1.45,3.19). Age was significant in association with past-year fall-related injury in the crude model, but the association attenuated to non-significance in the multivariable model. Having cataracts and an earth floor in the home were also significant in the crude but not the multivariable model. Generally the results of the weighted (Table 3) and unweighted multivariable regressions (Additional file 1: Appendix 1) were similar, except for cognition where *P*=0.012 in the multivariable weighted model and *P*=0.454 in the unweighted model.

**Table 3:** Crude and multivariable logistic regression of factors associated with past-year fall-related injury, adults aged 50 years and over, pooled countries, SAGE Wave 1, 2007–2010

	Crude models			Multivariable model		
Risk factor variables	Odds ratio	95 % CI	*P-value*	Odds ratio	95 % CI	*P-value*
Sex (reference: males)						
Female	1.73	1.43,2.10	*P*<0.0001	1.27	0.99,1.62	0.057
Age group (reference: 50–59 years)						
60–69	1.39	1.10,1.76	0.006	1.10	0.87,1.38	0.430
70–79	1.76	1.37,2.26	*P*<0.0001	1.23	0.98,1.54	0.079
80+	1.76	1.24,2.52	0.002	1.14	0.76,1.71	0.520
Residence (reference: urban)						
Rural	1.64	1.32,2.03	*P*<0.0001	1.36	1.06,1.75	0.017
Symptom-based depression (reference: no)						
Yes	3.62	2.87,4.56	*P*<0.0001	1.43	1.01,2.02	0.041
Chronic conditions (reference: none)						
One	2.03	1.71,2.41	*P*<0.0001	1.62	1.35,1.95	*P*<0.0001
Two or more	3.56	2.74,4.62	*P*<0.0001	2.15	1.45,3.19	*P*<0.0001
Cataracts (reference: no)						
Yes	1.93	1.55,2.40	*P*<0.0001	1.20	0.93,1.55	0.155
Sleep (reference: no problems sleeping)						
Severe or extreme problems sleeping	3.16	2.53,4.0	*P*<0.0001	1.54	1.15,2.08	0.004
Grip strength (average one unit change)	0.64	0.56,0.73	*P*<0.0001	0.87	0.75,1.00	0.056
Cognition (average one unit change)	0.78	0.73,0.89	*P*<0.0001	0.85	0.75,0.96	0.012
Water (reference: inside the home)						
Outside the home	2.05	1.69,2.49	*P*<0.0001	1.23	0.93,1.63	0.155
Flooring (reference: hard floor)						
Earth floor	2.09	1.67,2.61	*P*<0.0001	1.09	0.82,1.45	0.566
Country (reference: China)						
Ghana	0.83	0.64,1.07	0.146	0.54	0.39,0.74	*P*<0.0001
India	2.23	1.84,2.70	*P*<0.0001	1.01	0.78,1.30	0.962
Mexico	0.90	0.59,1.39	0.642	0.66	0.37,1.18	0.159
Russian Federation	0.81	0.54,1.20	0.293	0.70	0.44,1.12	0.139
South Africa	0.28	0.13,0.60	0.001	0.28	0.12,0.65	0.003

Pooled country weights applied. Multivariable model: VIF=1.019. LR chi-square (19)=483.18. Prob > chi-square=0.0000. Pseudo R-squared=0.0578

The results of the crude and multivariable regressions of past-year fall-related injury and disability are shown in Table 4. The unweighted results of the multivariable regressions are given in Additional file 1: Appendix 2, and the results are similar. In the crude model, disability was significantly higher by an average of 12.82 points in respondents who reported past-year fall-related injury. In the multivariable model, disability was on average 5.62 points higher for those who reported past-year fall-related injury. The association between past-year fall-related injury and disability attenuated after adjusting for sex, age, residence, chronic conditions, wealth and country of residence. In particular, older age (80+ years) and reporting having two or more chronic conditions were highly significant in association with disability.

**Table 4:** Crude and multivariable analysis of past-year fall-related injury and disability, adults aged 50 years and over, pooled countries, SAGE Wave 1, 2007–2010

	Crude models			Multivariable model		
Covariates	Coeff.	95 % CI	*P-value*	Coeff.	95 % CI	*P-value*
Fall-related injury (reference: none)						
Yes	12.82	10.45,15.20	*P*<0.0001	5.62	3.77,7.46	*P*<0.0001
Sex (reference: males)						
Female	4.57	3.94,5.20	*P*<0.0001	3.21	2.67,3.65	*P*<0.0001
Age group (reference: 50–59 years)						
60–69	5.07	4.30,5.85	*P*<0.0001	3.00	2.42,3.50	*P*<0.0001
70–-79	12.05	10.84,13.26	*P*<0.0001	8.88	8.03,9.73	*P*<0.0001
80+	22.43	20.40,24.45	*P*<0.0001	19.52	17.79,21.25	*P*<0.0001
Residence (reference: urban)						
Rural	5.69	4.46,6.92	*P*<0.0001	3.26	2.31,4.21	*P*<0.0001
Chronic conditions (reference: none)						
One	7.79	6.95,8.62	*P*<0.0001	4.76	4.12,5.40	*P*<0.0001
Two or more	19.55	17.91,21.19	*P*<0.0001	12.05	10.95,13.15	*P*<0.0001
Wealth quintile (reference: poor)						
Second poorest	−3.91	−5.20,-2.62	*P*<0.0001	−2.55	−3.51,–1.59	*P*<0.0001
Mid	−5.71	−7.08,-4.34	*P*<0.0001	−3.06	−3.98,–2.15	*P*<0.0001
Second highest	−8.29	−9.73,-6.84	*P*<0.0001	−4.21	−5.08,–3.33	*P*<0.0001
Highest (wealthiest)	−10.62	−12.14,–9.10	*P*<0.0001	−6.47	−7.56,–5.38	*P*<0.0001
Country (reference: China)						
Ghana	13.59	12.38,14.81	*P*<0.0001	11.13	10.01,12.26	*P*<0.0001
India	19.80	18.47,21.14	*P*<0.0001	16.59	15.43,17.75	*P*<0.0001
Mexico	6.43	4.38,8.47	*P*<0.0001	6.16	3.93,8.39	*P*<0.0001
Russian Federation	10.20	8.21,12.18	*P*<0.0001	7.22	5.34,9.11	*P*<0.0001
South Africa	11.43	10.05,12.81	*P*<0.0001	11.25	10.02,12.48	*P*<0.0001
R-squared				0.4443		
Constant				3.72	2.69,4.74	*P*<0.0001

Coeff.=coefficient. CI=confidence interval. Pooled country weights applied. Multivariable model: VIF=1.799. R-squared=0.4443

The age*fall-related injury interaction term was significant in association with disability (*P*<0.05). Table 5 shows the results of age-stratified regressions of past-year fall-related injury and disability, adjusting for sex, residence, chronic conditions, wealth and country as possible confounders. Past-year fall-related injury was highly significant (*P*<0.0001) in the 60–69 and 70–79 year age groups but less significant (*P*<0.05) in the 50–59 and 80 years and over age groups. Female sex was highly significant in all age strata (*P*<0.0001), as was the presence of one or more reported chronic conditions compared with no reported chronic conditions. In particular, respondents with two or more chronic conditions had disability scores that were, on average, almost 12 points higher than those with chronic conditions. The association between wealth and disability was strongest in the 50–59 year age group in which respondents with greater wealth had less disability. With the exception of the highest wealth quintile, the wealth gradient was not significant in the 80 years and older age group. However, the small sample size in this oldest age group is a possible reason for non-significance of the estimates.

**Table 5:** Multivariable analysis of past-year fall-related injury and disability stratified by age group, pooled countries, SAGE Wave 1, 2007–2010

	50–59 years		60–69 years		70–79 years		80+ years	
Covariates	Coeff.	95 % CI	Coeff.	95 % CI	Coeff.	95 % CI	Coeff.	95 % CI
Fall-related injury (reference: none)								
Yes	2.49^*^	0.38,4.60	**5.77**	**3.18,8.26**	**9.30**	**5.47,13.13**	11.65^*^	5.01,18.30
Sex (reference: males)								
Female	**2.92**	**2.31,3.54**	**3.11**	**2.20,4.02**	**3.13**	**1.89,4.36**	**5.18**	**2.55,7.82**
Residence (reference: urban)								
Rural	**2.27**	**1.16,3.37**	**2.83**	**1.74,3.93**	**4.53**	**2.82,6.24**	**9.35**	**6.29,12.42**
Chronic conditions (reference: none)								
One	**4.38**	**2.31,5.15**	**5.65**	**4.69,6.60**	**4.06**	**2.45,5.68**	**6.69**	**3.48,9.89**
Two or more	**11.15**	**9.50,12.79**	**12.54**	**10.93,14.16**	**11.82**	**10.03,13.61**	**14.68**	**10.50,18.87**
Wealth quintile (Reference: poor)								
Second poorest	−2.24^*^	−3.59,-0.89	−2.39^*^	−3.90,-0.87	−3.31^*^	−5.64,-0.98	−2.52	−6.13,1.09
Mid	**−2.93**	**−4.29,-1.57**	−2.80^*^	−4.42,-1.19	−3.45^*^	−5.51,-1.39	−3.37	−7.56,0.81
Second highest	**−3.50**	**−4.87,-2.12**	**−4.57**	**−6.17,-2.96**	**−6.26**	**−8.27,-4.26**	−2.84	−7.17,1.49
Highest (wealthiest)	**−5.58**	**−6.91,-4.26**	**−7.56**	**−9.30,-5.82**	**−9.01**	**−11.28,-6-73**	−5.85^*^	−10.29,-1.41
Country (reference: China)								
Ghana	**8.48**	**7.27,9.69**	**12.63**	**10.87,14.39**	**14.83**	**12.91,16.74**	**13.82**	**10.31,17.32**
India	**15.05**	**13.69,16.41**	**17.77**	**16.22,19.32**	**18.75**	**16.59,20.92**	**19.60**	**15.52,23.68**
Mexico	5.80^*^	1.89,9.72	**7.11**	**5.49,8.72**	5.37 ^*^	1.21,9.53	**9.23**	**5.30,13.16**
Russian Federation	**4.26**	**2.58,5.95**	**7.12**	**4.88,9.36**	**11.83**	**8.24,15.42**	**15.69**	**10.34,21.04**
South Africa	**9.35**	**7.97,10.74**	**12.99**	**10.90,15.08**	**14.16**	**10.91,17.40**	**14.33**	**9.19,19.47**
Constant	**4.81**	**3.44,6.18**	**6.46**	**4.82,8.10**	**11.95**	**9.87,14.04**	**14.52**	**11.26,17.78**
R-squared	0.3922		0.3910		0.3342		0.3318	

Coeff.=coefficient. CI=confidence interval. Pooled country weights applied. Bold= statistical significance *P<*0.0001. ^*^indicates 0.0001<*P<*0.05 statistical significance

## Discussion

Publications on falls epidemiology use various terms and definitions, making cross-country comparisons difficult. This is the first study of its kind to utilize nationally representative, comparable, population survey data to investigate past-year injury-related falls in older adults in China, Ghana, India, Mexico, the Russian Federation and South Africa. With population ageing proceeding rapidly in LMICs, there is more than ever a need for an evidence-based public health policy focus on falls prevention in older adults in these countries [9, 28]. The findings provide evidence of prevalence and risk factors associated with self-reported fall-related injury in older adults, and the extent to which fall-related injury affects disability in this group of six LMICs.

The prevalence of past-year fall-related injury in the SAGE countries was 6 %, which is in the same range as prevalence estimates reported for community-dwelling older adults in higher income countries [3, 4, 6]. According to the WHO, the burden of non-intentional injuries is disproportionately higher in developing countries [29]. Older adults are at higher risk of many types of injuries that can lead to death and disability, and falls are the most common cause of injury in older age groups [5]. Our results showed that about two-thirds of all past-year injuries in older adults in the six SAGE countries were fall-related.

Age is a common risk factor for falls [9, 12, 29, 53–55]. In this study of adults aged 50 years and over, age was significantly associated with past-year fall-related injury in the crude models. However, the effect of older age was tempered after adjusting for a wide range of risk factors in the multivariable model. Suggested reasons for this attenuation are possible under-reporting of fall-related injury in older age groups and the survivor effect, whereby potential respondents were removed as a result of fall-related mortality, thereby leaving a healthier, more robust cohort of older adults.

Female sex is widely reported in the literature as being associated with increased risk of falls in older age in many countries [23, 26, 54, 56]. The results of this study in the SAGE countries also showed that women were more likely to report past-year fall-related injuries and greater disability than men. It has been suggested that this may be due in part to higher fall-related mortality in men than in women and also to differences in bone density between older men and women [27, 57].

Environmental factors are predisposing conditions for fall-related injury [9]. Studies in developing countries show an increased risk of falls due to environmental factors such as open street gutters, poor quality footpaths and unsafe walking areas in rural areas [22, 28]. Respondents who lived in rural areas had higher odds of reporting past-year fall-related injuries in both the crude and multivariable models. It is also possible that the place of residence variable reflected socioeconomic factors. Having a water source outside the home and having earth flooring in the home were factors significantly associated with fall-related injury in the crude but not the multivariable model.

Although obesity has been associated with falls in some studies in high income countries [45], the literature reports differing patterns in developing countries. In a study conducted in an urban population in India for example, high BMI was protective for hip fracture [55]. It has been suggested that association between high BMI and falls is confounded by socioeconomic status [58] and also that the estrogenic effect of body fat on bone density in older women may be protective of fall-related injury [59]. Yet in contrast, other literature shows that most osteoporotic fractures occur in overweight and obese people [60]. We acknowledge that BMI measurement may not be the best method for determining obesity and may have been underestimated in the Asian respondents in this analysis [47, 61–63].

Sleep problems are common in older people, and there is evidence that poor sleep increases the risk of falls in older adults [24, 64–66]. The findings of this study of the six SAGE LMICs showed that respondents who reported having either severe or extreme problems sleeping were more likely to report past-year fall-related injuries.

Injuries sustained as a consequence of falls in older age are almost always more severe than those occurring in younger people. For injuries of the same severity, older people experience more disability, longer hospital stays, extended periods of rehabilitation and a higher risk of subsequent dependency [9]. These results further confirm evidence that falls are associated with increased disability even after adjusting for social and health-related factors as confounders [1, 27, 29, 45].

The results also support the assertion that fall-related injury is associated with greater disability and that these effects vary across age groups. Yet disability can be both a cause and a consequence of falls [12, 45], and this is further compounded by the fact that fall recurrence is high in older adults [67]. Given the cross-sectional study design, the proposition that greater disability leads to falls is equally valid [68]. Data from future waves of SAGE will provide information on the direction of the relationship and temporal associations.

At the population level, exercise programs that combine different aspects of balance, endurance, flexibility and strength can be relatively simple to deliver. Apart from preventing falls, they can confer other health benefits and should be an integral part of public health programs for older adults in all countries. Even if exercise does not prevent a fall, it has been shown to reduce injuries from falls [69]. At the individual level, a multi-factorial approach that tailors interventions to the profile of the individual and assesses all aspects of their condition may be more effective. Clinicians need to be trained to take many factors into account while delivering interventions in specialized settings [70].

The inclusion of a country dummy variable in the regressions highlights some country differences that can be followed up by policy-makers. For example, compared with China, the odds of past-year fall-related injury were higher in India but lower in the other four SAGE countries. In India, unintentional falls are a major public health problem that disproportionately affects older women [17, 71]. A study in rural India found that 38. 8 % of non-fatal injuries were due to falls, with one-third occurring in adults aged 60 years and over [54]. Yet studies of Chinese older adult populations consistently show a lower incidence of self-reported falls compared with older adults in Caucasian populations [12].

Hip fractures are a major public health problem for ageing populations. India is expected to experience a massive growth in hip fractures over coming decades [55, 72, 73]. In comparison, a population-based study of hip fractures amongst older residents of Beijing, China, showed that the rates were amongst the lowest in the world [74].

These SAGE results highlight differences between the world's two most populous countries, China and India. In China falls are not attracting public health attention to the same extent as they are in India, where falls in older adults are seen as an emerging public health problem in spite of widely varying reported prevalence rates [75]. A greater understanding of the differential impact of lifestyle and behavioural factors on falls in different populations and cultures is needed before the reasons for country differences can be unpacked and better understood.

### Strengths

The SAGE was conducted in six countries in a highly standardized manner. The questionnaire was first translated into the local language, and then back translated. All translations were validated before data collection commenced. Face-to-face interviews were administered to large representative samples of adult populations in LMICs from different geographic regions of the world. Every effort was made to ensure culturally appropriate interview settings. All interviewers were required to have participated in standardized training workshops of at least one week’s duration. The use of both household and individual level data allowed testing of biological, behavioural, environmental and socioeconomic covariates known to contribute to falls and fall-related injury. Data quality was closely monitored using strict quality assurance procedures. SAGE Wave 1 provides the first set of comparable nationally representative data for these six LMICs: China, Ghana, India, Mexico, the Russian Federation and South Africa.

The analysis includes the explicit definition of ‘past-year fall-related injury’ as the self-reported occurrence of any fall-related injury in the year prior to the survey interview. This precise standardized definition is used in all six SAGE countries. To our knowledge, other nationally representative definition-specific epidemiological falls data are not available in these or other developing countries.

While many of the determinants identified here are similar to those found in previous work, importantly this study also investigated other less studied factors, such as depression, multiple chronic conditions and cognition — all risk factors that require policy attention at an individual country level in LMICs. Locational factors such as rural residence and water availability are also proxies for poverty, which should be addressed by all countries with respect to the social determinants of health.

### Limitations

Recall and survivor bias can be limitations for epidemiological studies of adult populations. As already noted, survivor bias may help to explain the lack of effect of age and fall-related injury. Only 5 % of the pooled study population was aged 80 years and above, yet SAGE is one of the largest cohorts of oldest old available in LMICs. Cultural, contextual and structural factors may have differently affected the extent of under-reporting across the participating countries. The numbers of respondents who reported fall-related injuries within countries was relatively small, and the pooled analysis was undertaken to address small sample sizes. However, the pooling of country data to some extent masks patterns within individual countries.

The cross-sectional nature of the study presents limitations in terms of interpreting causal association. Respondents were asked whether they had had a fall in the previous 12 months, and it was not possible to differentiate between those who may have had single or multiple falls during this time period.

In spite of country differences in the reported prevalence of fall-related injury, consistent patterns emerge across countries in terms of the distribution by age, sex and other indicators. It is possible that systematic reporting differences are contributing to this variation. Other factors, such as the nature of the living environment or the risks related to the workplace, that may explain these differences, were not assessed in our study. Future waves of SAGE should examine the reasons for these variations in more detail.

## Conclusions

Morbidity and mortality resulting from fall-related injury is not widely recognized as a major public health problem in LMICs, possibly due to the lack of robust comparable data on risk factors and consequences. This study provides a much-needed platform for further epidemiological research in this area. While the prevalence of fall-related injuries might appear to be relatively low, the considerable morbidity and mortality associated with falls in older adults means that policy makers in LMICs need to be sensitized to the public health importance of this risk. Falls prevention strategies are not necessarily high cost. Given the rapid pace of population ageing in LMICs, encouraging exercise for older adults through health education programs and providing appropriate facilities for exercise in neighborhoods must become cost-effective policy priorities.

## Additional file

Additional file 1:
**Appendix 1 & 2.** Multivariable regressions: unweighted results.

## Abbreviations

BMI: body mass index
CI: confidence interval
Coeff: coefficient. Prob: probability
kg/m^2^: kilograms per square metre
LMICs: low- and middle-income countries
OR: odds ratio
SAGE: Study on global AGEing and adult health
VIF: variance inflation factor
WHO: World Health Organization
WHODAS 2:0: WHO Disability Assessment Schedule Version 2.0
YLD: years lived with disability
