# Supplementary material for: Prevalence, risk factors and disability associated with fall-related injury in older adults in low- and middle-incomecountries: results from the WHO Study on global AGEing and adult health (SAGE)
Source: BMC Med. 2015 Jun 23;13:147. doi: 10.1186/s12916-015-0390-8 (PMC4495610; doi:10.1186/s12916-015-0390-8)
Supplement: Additional file 1: — Appendix 1 & 2. Multivariable regressions: unweighted results. [file 12916_2015_390_MOESM1_ESM.docx]

| **APPENDIX 1. Multivariable logistic regression of factors associated with past-year fall-related injury, adults aged 50 years and over, pooled countries, SAGE Wave 1, 2007-2010, unweighted** | | | |
| --- | --- | --- | --- |
| **Risk factor variables** | Adjusted Odds Ratio | 95% CI | *p-value* |
| **Sex (Reference: males)** |  |  |  |
| Female | 1.35 | 1.16,1.58 | *p*<0.0001 |
| **Age Group (Reference: 50-59 years)** |  |  |  |
| 60-69 | 1.13 | 0.96,1.33 | 0.148 |
| 70-79 | 1.22 | 1.01,1.48 | 0.038 |
| 80+ | 1.52 | 1.16,1.99 | 0.002 |
| **Residence (Reference: urban)** |  |  |  |
| Rural | 1.36 | 1.16,1.60 | *p*<0.0001 |
| **Symptom-based depression (Reference: no)** |  |  |  |
| Yes | 1.53 | 1.24,1.89 | *p*<0.0001 |
| **Chronic Conditions (Reference: none)** |  |  |  |
| One | 1.46 | 1.25,1.71 | *p*<0.0001 |
| Two or more | 1.69 | 1.37,2.09 | *p*<0.0001 |
| **Cataracts (Reference: no)** |  |  |  |
| Yes | 1.25 | 1.04,1.51 | 0.016 |
| **Sleep (Reference: no problems sleeping)** |  |  |  |
| Severe or extreme problems sleeping | 1.53 | 1.27,1.84 | *p*<0.0001 |
| **Grip strength (Average one unit change)** | 0.93 | 0.84,1.02 | 0.102 |
| **Cognition (Average one unit change)** | 0.97 | 0.89,1.05 | 0.454 |
| **Water (Reference: inside the home)** |  |  |  |
| Outside the home | 1.28 | 1.07,1.53 | 0.007 |
| **Flooring (Reference: hard floor)** |  |  |  |
| Earth floor | 0.98 | 0.81,1.19 | 0.863 |
| **Country (Reference: China)** |  |  |  |
| Ghana | 0.68 | 0.52,0.87 | 0.002 |
| India | 1.34 | 1.10,1.63 | 0.003 |
| Mexico | 1.19 | 0.89,1.60 | 0.242 |
| Russian Federation | 0.79 | 0.61,1.03 | 0.079 |
| South Africa | 0.28 | 0.18,0.42 | *p*<0.0001 |

CI=confidence interval.

Coeff.=coefficient. CI=confidence interval.

| **APPENDIX 2. Multivariable analysis of past-year fall-related injury and disability, adults aged 50 years and over, pooled countries, SAGE Wave 1, 2007-2010, unweighted** | | | |
| --- | --- | --- | --- |
| **Covariates** | Coeff. | 95% CI | *p-value* |
| **Fall-related injury (Reference: none)** |  |  |  |
| Yes | 4.42 | 3.54,5.30 | *p*<0.0001 |
| **Sex (Reference: males)** |  |  |  |
| Female | 3.26 | 2.94,3.58 | *p*<0.0001 |
| **Age group (Reference: 50-59 years)** |  |  |  |
| 60-69 | 3.65 | 3.27,4.03 | *p*<0.0001 |
| 70-79 | 9.79 | 9.35,10.23 | *p*<0.0001 |
| 80+ | 19.29 | 18.62,19.95 | *p*<0.0001 |
| **Residence (Reference: urban)** |  |  |  |
| Rural | 2.20 | 1.85,2.55 | *p*<0.0001 |
| **Chronic conditions (Reference: none)** |  |  |  |
| One | 5.36 | 4.99,5.73 | *p*<0.0001 |
| Two or more | 12.25 | 11.75,12.75 | *p*<0.0001 |
| **Wealth quintile (Reference: poor)** |  |  |  |
| Second poorest | -1.60 | -2.11,-1.08 | *p*<0.0001 |
| Mid | -2.43 | -2.94,-1.91 | *p*<0.0001 |
| Second highest | -4.03 | -4.54,-3.52 | *p*<0.0001 |
| Highest (wealthiest) | -6.01 | -6.46,-5.49 | *p*<0.0001 |
| **Country (Reference: China)** |  |  |  |
| Ghana | 11.92 | 11.40,12.43 | *p*<0.0001 |
| India | 17.39 | 16.93,17.84 | *p*<0.0001 |
| Mexico | 6.91 | 6.23,7.59 | *p*<0.0001 |
| Russian Federation | 8.89 | 8.34,9.44 | *p*<0.0001 |
| South Africa | 10.00 | 9.45,10.55 | *p*<0.0001 |
| **R-squared** | 0.4443 |  |  |
| **Constant** | 2.49 | 1.93,3.06 | *p*<0.0001 |
